# Proximal femoral nail antirotation versus external fixation for unstable intertrochanteric fractures in elderly patients: A randomized controlled trial

**DOI:** 10.1097/MD.0000000000029384

**Published:** 2022-07-15

**Authors:** Yu Liang, Shaojing Liu, Lintao Li, Fenglin Zhong

**Affiliations:** a Department of Orthopaedics, Panzhihua Municipal Central Hospital, Province Sichuan, China.

**Keywords:** external fixation, hip, intertrochanteric fractures, proximal femoral nail antirotation

## Abstract

**Background:**

This study aimed to compare the clinical and radiographic outcomes of the proximal femoral nail antirotation (PFNA) and external fixation in the management of unstable intertrochanteric fractures in elderly patients.

**Methods:**

Eighty-seven of 114 patients with unstable intertrochanteric fractures were included in this study between January 2015 and June 2019, 46 were fixed with PFNA implant and 41 with external fixator. Patient baseline characteristics, functional and radiographic results, and postoperative complication were documented and compared between the 2 groups.

**Results:**

Prolonged operation duration, increased fluoroscopy time, and excess blood loss occurred in PFNA group. The functional results scores seemed higher in the PFNA than external fixation group in the first semester, and thereafter, there was no significant difference between groups. On early postoperative radiographs, better femur neck–shaft angle was acquired in the external fixators device, but the difference did not continue at final visit. The incidence rate of overall complications was 43.5% for the group PFNA and 100% for the group external fixation.

**Conclusions:**

Fewer postoperative complications occurred in PFNA than external fixator group when unstable intertrochanteric fractures were treated. Nevertheless, there was no significant difference detected in final functional and radiographic outcome between the 2 groups.

## 1. Introduction

The incidence of intertrochanteric fractures has been increasing year by year because of the aggravation of population aging, which is usually accompanied by high comorbidity and mortality. Surgery is often preferred allowing early mobilization as soon as possible. Many studies have proved that proximal femoral nail antirotation (PFNA) can be used safely in unstable intertrochanteric fractures in elderly patients,^[1–3]^ while some other authors have reported that external fixation (EF) could also successfully treat the majority of unstable intertrochanteric fractures in these patients,^[4–7]^ both PFNA and EF are minimally invasive surgical methods. Nevertheless, there is no relevant study that compares PFNA with EF in the treatment of intertrochanteric fractures (AO/OTA Type A2.2-A.3.3) in terms of functional and radiographic outcomes in gerontal patients reported in the literature so far. Therefore, conclusive data regarding which implant is superior are lacking in this area.

The hypothesis tested in this study was that there is no difference between PFNA and EF in AO/OTA A2.2-A.3.3 intertrochanteric fractures.

## 2. Methods

**Figure 1.**
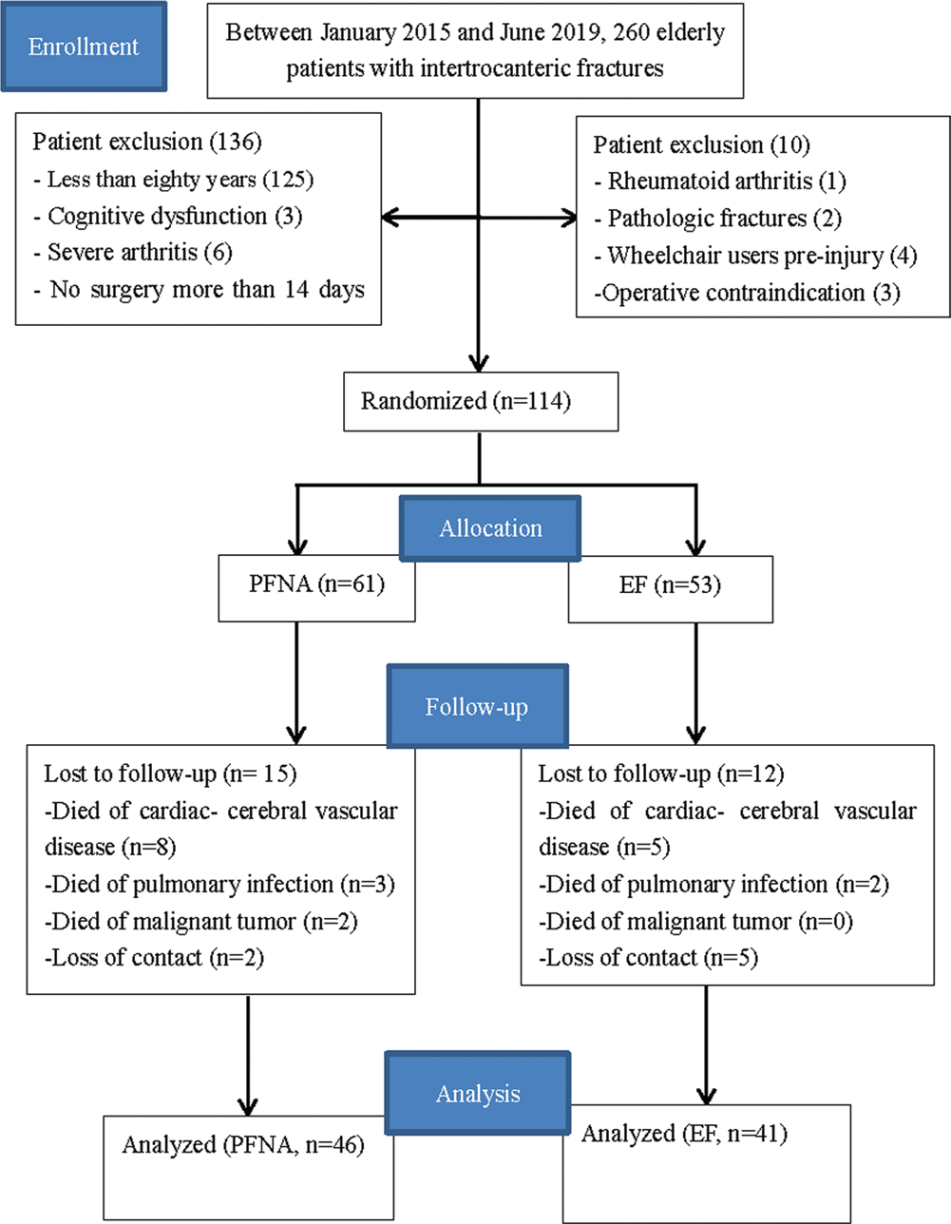
Flowchart of patients selected through the trial. EF = external fixation, PFNA = proximal femoral nail antirotation.

From January 2015 to June 2019, 260 patients with intertrochanteric fractures admitted to our department were considered for inclusion in this study. Inclusion criteria were an age of 80 years or older, an unstable intertrochanteric fracture (AO/OTA 31.A2.2-31.A3.3), medical fitness for surgery, and a fracture that had occurred <2 weeks before the time of enrollment. The exclusion criteria were pathologic fractures, bilateral fractures, an inability to walk before the fracture, severe dementia, limited life expectancy due to substantial medical comorbidities, an inability to comply with rehabilitation or to complete the forms, and a previous operation in the same hip/femur. Eligible patients were enrolled for at least 1-year period on the basis of the above inclusion criteria (as shown in Fig. 1).

This prospective randomized trial was approved by the Panzhihua Municipal Central Hospital Institutional Review Board. Study design and reporting were based on the CONSORT principles.^[8]^ Patients with an unstable intertrochanteric fracture were identified by 2 consultants on admission to the emergency department, if they met the inclusion criteria, and informed consent was obtained, the patients were scheduled for surgery. Permuted block randomization was applied to designate the 2 treatment methods. Sealed envelopes involving the randomly generated method were opened just prior to surgery, and the designated procedure was then administrated. The clinical follow-up estimate was implemented by qualified researcher who had gained all of the patient files and documents in our department. The radiographic result was assessed by a single independent orthopedic surgeon.

### 2.1. Patient baseline characteristics

Patient demographics at the time of injury, including age, gender, height, weight, marital status, smoking and drinking history, concomitant medications, and existing injuries or disorders of the ipsilateral leg were documented. The baseline injury characteristics including the type of accident, level of energy of the injury, fracture type according to the AO/OTA classification as well as ASA classification,^[9]^ and injury–operation interval were also recorded.

### 2.2. Surgical procedure and postoperative management

Spinal anesthesia were applied in all patients; the surgical procedure for implantation of the PFNA and EF devices included using a traction table with an attempt at closed reduction with fluoroscopic guidance. Thereafter, the procedures vary substantially for the different implants. In the PFNA group, a guidewire was inserted through a small incision proximal to the greater trochanter under image intensifier; the trochanter was then drilled and the medullary canal may be reamed. The nail was then inserted and fixed into the femoral head with a helical blade. The nail was then locked distally using a guide arm; the surgical drain was not installed under the wound in all patients. In the EF group, patients were placed on fracture table and closed reduction was administrated under fluoroscopy; furthermore, 2 long, self-tapping, and hydroxyapatite-coated 6.5-mm Schanz screws were first inserted and advanced to within 10 mm from the subchondral bone of the head. Then, a unilateral, external fixator frame with uncoated stainless steel pins, which attached to the proximal pins, acted as a template for the introduction of the 2 distal pins. In all cases, closed reduction of the fractures was achieved and no open reduction was performed. The transfusion criterion was a hemoglobin concentration of <8 g/dL. Duration of the surgical procedure and fluoroscopy usage were recorded. The duration of surgery was noted in concordance with the records of the anesthesiology team and included closed reduction of the fracture. Fluoroscopy use was saved after the procedure according to the frequency on the screen of the fluoroscopy device.

Isometric quadriceps and mechanical calf pumps were immediately initiated after surgery. Subcutaneous enoxaparin sodium injections were used so as to prevent deep venous thrombosis; all patients were advised to sit on bed or on a chair on the first postoperative day, and active hip movements were also started under the supervision and guidance of physiotherapists.

### 2.3. Clinical and radiological assessment

Operating duration, fluoroscopy time, transfused blood units, duration of hospitalization, complications, healing time, and mortality were documented until the final follow-up evaluation. The primary functional outcome tools used in this study were the Harris Hip Score^[10]^ and the 36-item Short-Form Health Survey (SF-36). The HHS was considered excellent (90–100), good (80–89), fair (70–79), or poor (<70); the latter containing a direct quantitative indication of quality of life was used to assess the condition of physical and mental health in elderly patients.^[11]^ Additionally, visual analog scale (VAS) was also specifically assessed by asking the patient to rate his or her level of pain in the affected limb using a numeric rating scale. The study protocol required examinations at 1 month, 3 months, 6 months, and 1 year. At each visit, patients were also requested to complete the domestic SF-36.^[12]^

Anteroposterior and lateral radiographs were evaluated for the quality of fracture reduction according to the classification described by Baumgaertner et al.^[13]^ The reduction was considered good when normal or slight valgus alignment was evident on the anteroposterior radiograph, <20° of angulation on the lateral radiograph, and no >4 mm of displacement of any fragment. An acceptable reduction was characterized by a reduction that met the criterion of a good reduction with respect to either alignment or displacement, but not both. Finally, a poor reduction did not meet any of the above criteria. We also assessed the trend of the femoral neck–shaft angle postoperatively based on anteroposterior radiographs of the affected hip during follow-up. Besides, implant complications (including breakage, loosening, cut/back out), loss of reduction, prosthetic instability, periprosthetic fracture, lateral cortex fracture, delayed union, malunion, or nonunion were also noted. On the first postoperative day, operated-side anteroposterior and lateral hip radiographs were taken for each patient, and the same radiographs were obtained at 1, 3, 6, and 12 months postoperatively.

### 2.4. Statistical analysis

Continuous variables are presented with mean and standard deviation or with median and interquartile range. For comparison of proportions, chi-square and Fisher exact tests were used. For comparison of study variables between groups, nonparametric Mann–Whitney test was computed for nonnormal variables and Student *t* test for normal variables. Statistical significance was set at a *P* value of .05, and analyses were conducted using SPSS statistical software (version 22.0).

## 3. Results

Eighty-seven patients completed the final follow-up except for 27 who were not available because of various variability; 20 patients died (12 due to cardiac–cerebral vascular accident, 6 due to pulmonary infection, and 2 due to malignant tumor) and 7 lost contact. Therefore, the trial consisted of 87 patients (46 in the PFNA group and 41 in the EF group). There was no significant difference in demographics and clinical characteristics of the 2 study groups such as age, sex, side of fracture, mechanism of injury, concomitant disease, and preoperative ASA classification and the detailed information are presented in Table 1.

**Table 1:** Patient baseline characteristics.

	**PFNA**		**EF**		
**Factors**	**n (%**)	**Mean** ± **SD**	**n (%) mean**	**Mean** ± **SD**	***P* value**
Age (yr)	46 (100)	86.1 ± 3.5	41 (100)	85.6 ± 3.2	.531
Sex					
Male	19 (41)		13 (32)		.354
Female	27 (59)		28 (68)		
Etiology					
Fall	43 (93)		38 (93)		.220
Fall from height	3 (7)		1 (2)		
Motor vehicle accident	0 (0)		2 (5)		
Marital status					
Married	13 (28)		14 (34)		.816
Separated	3 (7)		2 (5)		
Widowed	30 (65)		25 (61)		
Living with family					
Yes	40 (87)		37 (90)		.886
No	6 (13)		4 (10)		
Body mass index (kg/m^2^)	46 (100)	27.6 ± 2.05	41 (100)	27.1 ± 1.99	.245
Smoking status					
Yes	0 (0)		0 (0)		.855
Cessation	4 (8.7)		5 (12.2)		
No	42 (91.3)		36 (87.8)		
Diabetic patients	10 (21.7)		8 (19.5)		.798
Fracture type (AO/OTA)					
31 A2	20 (43.5)		19 (46.3)		.789
31 A3	26 (56.5)		22 (53.7)		
Osteoporosis					
Yes	46 (100)		41 (100)		1.000
No	0 (0)		0 (0)		
Complication					
Yes	44 (95.7)		40 (97.6)		1.000
No	2 (4.3)		1 (2.4)		
Preinjury SF-36 Physical Health (0–100)	40 (97.6)	40.48 ± 11.8	43 (93.5)	41.25 ± 12.1	.769
Preinjury SF-36 Mental Health (0–100)	40 (97.6)	42.33 ± 13.7	43 (93.5)	42.3 ± 15.36	.994
Injury–operation interval (d)	46 (100)	4.28 ± 1.24	41 (100)	4.48 ± 1.27	.448
ASA classification					
P2	26 (56.5)			22 (53.7)	.962
P3	16 (34.8)			15 (36.6)	
P4	(8.7)			(9.8)	

AO/OTA = Arbeitsgemeinschaft fur osteosynthesefragen/Orthopaedic Trauma Association, ASA = American Society of Anesthesiologists, EF = external fixation, N = number, P = physical status, PFNA = proximal femoral nail antirotation, SD = standard deviation,

SF-36 = 36-item Short-Form Health Survey.

### 3.1. Clinical results

The median operative time during PFNA surgery was 52.7 ± 16.2 minutes (range, 30–95 minutes), which was significantly more than the median time of 38.4 ± 5.8 minutes (range, 29–51 minutes) required for EF surgery (*P* = .000). The median fluoroscopy time was 65.3 ± 17.1 seconds (range, 35–115 seconds) in group PFNA and 49.9 ± 10.8 seconds (range, 26–71 seconds) in group EF (*P* = .000). Hemoglobin <9 g/dL was observed in 37% of patients in group PFNA (17 patients) and in 27% of patients in group EF (11 patients; *P* = .247); furthermore, blood transfusion was required in 8.7% of patients in group PFNA (4 patients) and in 2.4% of patients in group B (1 patient; *P* = .429); the former 2 patients had to be hospitalized again and treated owing to implant failure that needed to be transfused. There was significant difference in the amount of blood loss between PFNA and EF-treated patients (105.3 ± 49.5 vs 11.0 ± 4.0 mL, *P* = .000). The median hospitalization time was 4.8 ± 1.1 days (range, 4–8 days) in group PFNA and 4.4 ± 1.0 days (range, 3–8 days) in group EF (*P* = .087). The average time to union was 12.57 ± 1.07 weeks (range, 10–14 weeks) in group PFNA and 12.66 ± 1.00 weeks (range, 11–15 weeks) in group EF (*P* = .675).

With regards to functional outcome scores, there existed different tendency between the 2 groups during the follow-up. The HHS was greater in the PFNA than EF group in the first semester, and thereafter, there was no significant difference between groups at final visit. Finally, <17% of the patients with osteoporosis had satisfactory functional scores at the final follow-up: none had excellent scores, 14 had good scores, 58 had fair scores, and 15 had poor scores (Table 2). Although there was steady improvement in the SF-36 physical and mental health over the 12-month period, the scores did not return to their preinjury level in either the PFNA or the EF group. At 1 and 3 months postoperatively, the PFNA group had significantly better daily activities than the EF group, as defined by SF-36 physical health (*P* = .000, *P* = .000, respectively), while there was no significant difference in the SF-36 mental health scores between groups at 1, 3, and 12 months postoperatively but not at 6 months. From the first to the third month, the VAS score was significantly higher in the EF than in the PFNA group, and whereafter, the result was just the opposite (Table 2).

**Table 2:** Comparison of functional outcomes between study groups.

	**PFNA**	**EF**	
**Variable/time point**	**Mean** ± **SD**	**Mean** ± **SD**	P value
Functional outcomes			
Harris Hip Score			
1 month	59.72 ± 5.05	41.00 ± 8.02	.000
3 months	66.02 ± 5.89	53.37 ± 9.35	.000
6 months	73.07 ± 5.16	67.10 ± 7.29	.000
12 months	75.50 ± 4.99	74.56 ± 5.89	.423
SF-36 Physical Health			
1 month	25.17 ± 3.93	20.93 ± 5.14	.000
3 months	27.87 ± 3.54	23.34 ± 4.65	.000
6 months	31.28 ± 3.63	29.15 ± 4.40	.015
12 months	35.24 ± 2.79	34.80 ± 4.18	.576
SF-36 Mental Health			
1 month	27.15 ± 5.0	25.90 ± 4.29	.217
3 months	29.35 ± 4.66	27.80 ± 3.93	.101
6 months	32.52 ± 4.28	30.85 ± 3.3	.048
12 months	35.93 ± 4.72	34.07 ± 4.10	.054
VAS score			
1 month	2.22 ± 1.11	4.51 ± 0.90	.000
3 months	2.04 ± 0.97	4.29 ± 0.68	.000
6 months	1.47 ± 0.94	1.05 ± 0.63	.013
12 months	1.30 ± 0.96	0.39 ± 0.49	.000

EF = external fixation, PFNA = proximal femoral nail antirotation, SD = standard deviation, SF-36 = 36-item Short-Form Health Survey, VAS = visual analog scale.

### 3.2. Radiographic outcomes

At the 12-month follow-up visit, there was no difference between groups with regard to reduction quality of intertrochanteric fractures (Table 3). Similarly, no significant differences were observed in any cases with loss of fracture reduction when final time point was compared with the first one in both groups (*P* = .739, *P* = .242, respectively). Nevertheless, significant differences in femur neck–shaft angle were discovered between the groups on the first day and at 1 month except at 3, 6, and 12 months postoperatively. Besides the changes from the first day to 12 month, both the PFNA and EF groups had reached statistical significance in femur neck–shaft angle (paired *t* test, *P* = .000, *P* = .000, respectively).

**Table 3:** Comparison of radiographic measurements between treatment groups.

	PFNA		EF		
**Variable/time point**	**n (%**)	**Mean** ± **SD**	**n (%**)	**Mean** ± **SD**	***P* value**
Reduction quality					
First day postoperatively					
Good	28 (60.9)		26 (63.4)		.753
Acceptable	14 (30.4)		10 (24.4)		
Poor	4 (8.7)		5 (12.2)		
1 month					
Good	26 (56.5)		25 (61)		.892
Acceptable	12 (26.1)		9 (22)		
Poor	8 (17.4)		7 (17)		
3 months					
Good	25 (54.3)		23 (56.1)		.910
Acceptable	13 (28.3)		10 (24.4)		
Poor	8 (17.4)		8 (19.5)		
6 months					
Good	25 (54.3)		23 (56.1)		.230
Acceptable	15 (32.6)		8 (19.5)		
Poor	6 (13.1)		10 (24.4)		
12 months					
Good	25 (54.3)		21 (51.2)		.219
Acceptable	15 (32.6)		9 (22)		
Poor	6 (13.1)		11 (26.8)		
Femur neck–shaft angle (°)					
First day postoperatively		134 ± 2.20		136.37 ± 1.59	.000
1 month		133.33 ± 2.37		135.54 ± 1.36	.000
3 months		131.04 ± 7.71		133.34 ± 3.31	.081
6 months		133.09 ± 2.58		132.71 ± 3.99	.605
12 months		131.87 ± 2.54		131.17 ± 3.82	.325

EF = external fixation, N = number, PFNA = proximal femoral nail antirotation, SD = standard deviation.

### 3.3. Complications

The incidence of overall complications was 43.5% for the group PFNA and 100% for the group EF (Table 4). This difference was mainly attributed to an increased incidence of local pain or irritation in the entrance of the screws in the EF group of patients. The most common problems were blade back out and local pain in the PFNA group while local pain in the EF group. Other usual problems encountered intraoperatively or postoperatively were technical problems and lateral cortex fracture in the PFNA group; on the contrary, screw cut-out, loss of reduction, malunion, superficial pin-site infection, and lower limb shortening were usually encountered in the EF group. Two cases in the PFNA group and 3 cases in the EF group were considered implant failures, the former had to undergo additional surgical procedure due to blade back out and cut out, while the latter just agreed to remove their EF and further surgical intervention were refused because of fears caused by surgery. Then, restricted weight bearing was carried out and walking hip braces were worn until callus formation was obviously seen.

**Table 4:** List of all complications for PFNA and EF groups.

	**PFNA(N = 46), n**	**%**	**EF(N = 41), n**	**%**	***P* value**
All complications	20	43.5	41	100	.000
Intraoperative complication	5	10.9	0	0	.087
Technical problem	5	10.9	0	0	.087
Postoperative complication	15	32.6	41	100	.000
Implant loosening	0	0	5	12.2	.048
Blade or screw cut out	4	8.7	7	17.1	.241
Blade or screw back out	10	21.7	0	0	.005
Loss of reduction	4	8.7	8	19.5	.144
Delayed healing	3	6.5	5	12.2	.567
Malunion	2	4.3	10	24.4	.007
Nonunion	0	0	0	0	1.000
Prosthetic instability	1	2.2	0	0	1.000
Periprosthetic fracture	0	0	0	0	1.000
Superficial pin-site infection	0	0	21	51.2	.000
Deep wound infection	0	0	0	0	1.000
Local pain or irritation	10	21.7	41	100	.000
Lateral cortex fracture	5	10.9	0	0	.087
Lower limb shortening (>1 cm)	1	2.2	0	14.6	.082
Avascular necrosis of femoral head	0	0	0	0	1.000
Implant failure	2	4.3	3	7.3	.895
Revision surgery	2	4.3	0	0	.496

EF = external fixation, N = number, PFNA = proximal femoral nail antirotation.

## 4. Discussion

Despite numerous operative techniques and fixation devices, no 1 method of treatment has gained universal acceptance for the treatment of unstable intertrochanteric hip fractures. Currently, it is widely accepted that the treatment of intertrochanteric fractures in geriatrics is to offer rigid fixation at fracture site, allowing early mobilization as soon as possible so as to reduce complications and mortality.

In the present study, the clinical results showed important differences in the aspect of operating duration, fluoroscopy time, and blood loss, while blood transfusion, hospitalization time, and time to union were not included. It was probable that better explanation was good reduction should be first performed in the PFNA group because nonanatomical reduction inevitably leads to intramedullary nailing incorrect insertion or implant malpositioning, and which was closely related to mechanical failure of internal fixation of extracapsular proximal femoral fracture.^[14]^ On the contrary, to some extent, malreduction was acceptable as long as stable fixation of fracture fragments was acquired when applying external fixator. Hence, decreased operating duration and fluoroscopy time were achieved in EF group. Massive blood loss could aggravate functional outcomes and increase mortality in patients with hip fracture by lowering hemoglobin levels, D’ArrigoCarmelo et al[AQ: Please note that the reference “Carmelo et al” is not listed in the reference list. Please add it to the list or delete the citation.]^[15]^ had reported that the mean visible blood loss was 226 mL in the PFNA group observed intraoperatively in the treatment of intertrochanteric fractures, whereas 105 mL in our study. The loss of blood mainly occurred in the procedure of the medullary canal was extensively reamed because of the large diameter of the proximal aspect of the implant.

Biomechanically, although intramedullary nail is the strongest option among osteosynthesis materials,^[16,17]^ there were no significant differences between the intramedullary and extramedullary treatment groups with regard to the clinical outcome,^[18]^ which was consistent with our study that there was no statistical difference between PFNA and EF groups in terms of postoperative final clinical function, as assessed with HHS and SF-36 Physical Health at the different time points, even though the HHS and SF-36 Physical Health scores were higher in the PFNA than EF group in the first semester. Some reasons may be attributed to this phenomenon. First, the configuration of the external fixator carried by patients brought a lot of inconvenience in their daily living activities, especially for function of bathing and dressing. Second, the irritation or pain of pin site had a bad effect on patient’s resting state. Finally, all external fixators had been completely removed at 6 months postoperatively and further bad influence related to external fixators might not continue any more, so that no significant difference existed between the 2 study groups at final follow-up. Similarly, from 1 month to 3 months, the VAS scores were greater in the EF group than in the PFNA group, and since then, the result was totally reversed, which was consistent with Kim et al^[19]^ had considered that inferior VAS score was usually generated as nail protrusion over the greater trochanter area occurs frequently after the surgical treatment of intertrochanteric fracture using PFNA. What is more, the SF-36 Physical and Mental Health scores at the 12-month time point were significantly lower than the initial prefracture values, indicating overall loss of function and confidence after the fracture regardless of the treatment modality employed, which are similar to those found in the literature.^[18,20,21]^

The evaluation index of radiographic outcomes in this paper was reduction quality and femur neck–shaft angle. Notwithstanding no significant differences were demonstrated in terms of reduction quality between groups, the variation trend of femur neck–shaft angle showed different results. As could be seen from Table 3, the change of femoral neck–shaft angle was more greater in the EF group than the PFNA group, that is, the use of the PFNA could lead to better radiographic outcomes, which was similar to the result of Reindl et al^[18]^; compared to extramedullary fixation, intramedullary devices had improved radiographic outcomes in their study.

Intraoperative complication more usually occurred in PFNA than in the EF group because good reduction was obtained before surgery in some cases but was then lost when the nail was inserted; this perspective was in accordance with Seyhan et al^[22]^ who stated that the most time-consuming step was the definition of the nail entry point since correct positioning of the entry point was crucial for the correct placement of the implant. However, the PFNA group seemed superior in terms of complications that occured after surgery. Pin tract infection and local irritation were problems inherent with the use of EF. In addition, more complications might be correlated with mechanical weakness of the EF devices because posteromedial buttress was missing in all treated cases, which would lead to the implant that is subjected to severe bending stress, and the loads applied are concentrating on the weakest medial side, so high axial bending forces might predispose the varus collapse of the fracture and implant failure, especially when patient was allowed to mobilize postoperatively as early as possible. Therefore, as a result of the longer lever arm compared with intramedullary devices, the applied device might suffer higher incidence of postoperative complications. The result confirmed with foregoing clinical findings of using external fixator in the management of unstable intertrochanteric fractures with higher complication rate.^[5]^

There are several limitations to our study. First, the number of patients participated is small and the differences between PFNA and EF are possibly minimal so enough sample is needed to perceive delicate difference. Second, the follow-up could be considered as being short because it was limited to 1 year. This is a usual problem encountered in previous studies. It is partially associated with the advanced age of the patients because most of them could not take part in the follow-up by themselves. Besides, the mortality in the first year after intertrochanteric fracture is rather high. Hence, the longer-term follow-up in these patients was challenging due to loss to follow-up bias and high mortality.^[23,24]^

In a nutshell, this study regarding intertrochanteric fracture treatment modalities did not clearly favor 1 implant over another; even though PFNA group led to significantly fewer postoperative complications, this was not equal to a significant difference in functional outcome and radiographic outcome, respectively.

## Acknowledgments

The authors gratefully thank those surgeons and patients who made this study possible.

## Author contributions

Conceptualization: Yu Liang.

Data curation: Shaojing Liu, Lintao Li.

Formal analysis: Lintao Li, Fenglin Zhong.

Investigation: Shaojing Liu.

Methodology: Yu Liang, Fenglin Zhong.

Software: Shaojing Liu,.

Writing – original draft: Yu Liang.

## References

[1] Simmermacher RK Ljungqvist J Bail H; AO - PFNA Studygroup. The new proximal femoral nail antirotation (PFNA) in daily practice: results of a multicentre clinical study. Injury. 2008;39:932–9. 18582887 10.1016/j.injury.2008.02.005

[2] Sahin S Ertürer E Oztürk I. Radiographic and functional results of osteosynthesis using the proximal femoral nail antirotation (PFNA) in the treatment of unstable intertrochanteric femoral fractures. Acta Orthop Traumatol Turc. 2010;44:127–34. 20676015 10.3944/AOTT.2010.2237

[3] Soucanye de Landevoisin E Bertani A Candoni P. Proximal femoral nail antirotation (PFN-ATM) fixation of extra-capsular proximal femoral fractures in the elderly: retrospective study in 102 patients. Orthop Traumatol Surg Res. 2012;98:288–95. 22483629 10.1016/j.otsr.2011.11.006

[4] Kourtzis N Pafilas D Kasimatis G. Management of pertrochanteric fractures in the elderly patients with an external fixation. Injury. 2001;32(Suppl 4):SD115–28. 11812485 10.1016/s0020-1383(01)00158-9

[5] Petsatodis G Maliogas G Karikis J. External fixation for stable and unstable intertrochanteric fractures in patients older than 75 years of age: a prospective comparative study. J Orthop Trauma. 2011;25:218–23. 21399471 10.1097/BOT.0b013e3181e9378a

[6] Parker MJ Das A. Extramedullary fixation implants and external fixators for extracapsular hip fractures in adults. Cochrane Database Syst Rev. 2013;2013:CD000339. 23450528 10.1002/14651858.CD000339.pub3 PMC7061252

[7] Yousry AH Chotai PN El Ghazaly SA. Outcomes of trochanteric external fixation for geriatric inter-trochanteric hip fractures. J Orthop. 2015;12:174–8. 26566315 10.1016/j.jor.2015.05.019 PMC4602004

[8] Moher D Schulz KF Altman DG; CONSORT GROUP (Consolidated Standards of Reporting Trials). The CONSORT statement: revised recommendations for improving the quality of reports of parallel-group randomized trials. Ann Intern Med. 2001;134:657–62. 11304106 10.7326/0003-4819-134-8-200104170-00011

[9] Owens WD Felts JA Spitznagel EL Jr. ASA physical status classifications: a study of consistency of ratings. Anesthesiology. 1978;49:239–43. 697077 10.1097/00000542-197810000-00003

[10] Harris WH. Traumatic arthritis of the hip after dislocation and acetabular fractures: treatment by mold arthroplasty. An end-result study using a new method of result evaluation. J Bone Joint Surg Am. 1969;51:737–55. 5783851

[11] Jordan-Marsh M Cody M Silverstein M. Assessing a self-report health measure for non-English-speaking elders: issues in using the SF36 Health Survey. Research on Social Work Practice. 2007;18:55–65.

[12] Zhou B Chen K Wang JF. Reliability and validity of a Short-Form Health Survey Scale (SF-36), Chinese version used in an elderly population of Zhejiang province in China [in Chinese]. Zhonghua Liu Xing Bing Xue Za Zhi. 2008;29:1193–8. 19173962

[13] Baumgaertner MR Curtin SL Lindskog DM. The value of the tip-apex distance in predicting failure of fixation of peritrochanteric fractures of the hip. J Bone Joint Surg Am. 1995;77:1058–64. 7608228 10.2106/00004623-199507000-00012

[14] Morvan A Boddaert J Cohen-Bittan J. Risk factors for cut-out after internal fixation of trochanteric fractures in elderly subjects. Orthop Traumatol Surg Res. 2018;104:1183–7. 30342858 10.1016/j.otsr.2018.06.021

[15] D’Arrigo C Carcangiu A Perugia D. Intertrochanteric fractures: comparison between two different locking nails. Int Orthop. 2012;36:2545–51. 23104674 10.1007/s00264-012-1684-5 PMC3508047

[16] Parker MJ Handoll HH. Gamma and other cephalocondylic intramedullary nails versus extramedullary implants for extracapsular hip fractures in adults. Cochrane Database Syst Rev. 2008;CD000093. 16235272 10.1002/14651858.CD000093.pub3

[17] Jacobs RR McClain O Armstrong HJ. Internal fixation of intertrochanteric hip fractures: a clinical and biomechanical study. Clin Orthop Relat Res. 1980;146:62–70. 7371270

[18] Reindl R Harvey EJ Berry GK. Canadian Orthopaedic Trauma Society (COTS). Intramedullary versus extramedullary fixation for unstable intertrochanteric fractures: a prospective randomized controlled trial. J Bone Joint Surg Am. 2015;97:1905–12. 26631990 10.2106/JBJS.N.01007

[19] Kim SS Kim HJ Lee CS. Clinical outcomes of PFNA-II in the Asian intertrochanteric fracture patients: comparison of clinical results according to proximal nail protrusion. Injury. 2020;51:361–6. 31812322 10.1016/j.injury.2019.11.040

[20] Vaquero J Munoz J Prat S. Proximal femoral nail antirotation versus gamma3 nail for intramedullary nailing of unstable trochanteric fractures. A randomised comparative study. Injury. 2012;43(Suppl 2):S47–54. 23622992 10.1016/S0020-1383(13)70179-7

[21] Aktselis I Kokoroghiannis C Fragkomichalos E. Prospective randomised controlled trial of an intramedullary nail versus a sliding hip screw for intertrochanteric fractures of the femur. Int Orthop. 2014;38:155–61. 24318319 10.1007/s00264-013-2196-7 PMC3890147

[22] Seyhan M Turkmen I Unay K. Do PFNA devices and Intertan nails both have the same effects in the treatment of trochanteric fractures? a prospective clinical study. J Orthop Sci. 2015;20:1053–61. 26197959 10.1007/s00776-015-0750-4

[23] Zhang H Zeng X Zhang N. INTERTAN nail versus proximal femoral nail antirotation-Asia for intertrochanteric femur fractures in elderly patients with primary osteoporosis. J Int Med Res. 2017;45:1297–309. 28587540 10.1177/0300060517710584 PMC5625524

[24] Nazrun AS Tzar MN Mokhtar SA. A systematic review of the outcomes of osteoporotic fracture patients after hospital discharge: morbidity, subsequent fractures, and mortality. Ther Clin Risk Manag. 2014;10:937–48. 25429224 10.2147/TCRM.S72456 PMC4242696

